# Spectral CT Analysis of Solitary Pulmonary Nodules for Differentiating Malignancy from Benignancy: The Value of Iodine Concentration Spatial Distribution Difference

**DOI:** 10.1155/2018/4830659

**Published:** 2018-12-09

**Authors:** Linyu Wu, Guoquan Cao, Liang Zhao, Kun Tang, Jie Lin, Shouliang Miao, Tingting Lin, Jieke Sun, Xiangwu Zheng

**Affiliations:** Department of Radiology, The First Affiliated Hospital of Wenzhou Medical University, Wenzhou 325035, China

## Abstract

**Objective:**

The objective is to assess the value of spatial distribution difference in iodine concentration between malignant and benign solitary pulmonary nodules (SPNs) by analyzing multiple parameters of spectral CT.

**Methods:**

Sixty patients with 39 malignant nodules and 21 benign nodules underwent chest contrast CT scans using spectral imaging mode during pulmonary arterial phase (PP), arterial phase (AP), and venous phase (VP). Iodine concentrations of proximal and distal regions in pulmonary nodules on iodine-based material decomposition images were recorded. Normalized iodine concentration (NIC) and the differences in NIC between the proximal and the distal regions (dNIC) were calculated. The two-sample t-test and Mann–Whitney U-test were performed to compare the multiple parameters generated from spectral CT between malignant and benign nodules. Receiver operating characteristic (ROC) curves were generated to calculate sensitivity and specificity.

**Results:**

NIC in the proximal region (NIC_pro_) and NIC in the distal region (NIC_dis_) between malignant and benign nodules at AP (NIC_pro_, P=0.012; NIC_dis_, P=0.024), and VP (NIC_pro_, P=0.005; NIC_dis_, P =0.004) were significantly different. NIC_pro_ at PP (P = 0.037) was also found significantly different between malignant and benign nodules; however, no significant differences were found in NIC_dis_ at PP (P = 0.093). In addition, the dNIC of malignant nodules was significantly higher than that of benign ones at PP (median and interquartiles (0.31, 0.11, 0.57 versus -0.26, -0.5, -0.1); p≤0.001), AP (mean dNIC, 0.093 ±0.094 versus -0.075±0.060; p≤0.001), and VP (mean dNIC, 0.171±0.137 versus -0.183±0.127; p≤0.001). The sensitivity and specificity (93%, 95%, respectively) of dNIC during VP were higher than other parameters, with a threshold value of -0.07.

**Conclusions:**

Spectral CT imaging with multiple parameters such as NIC_pro_, NIC_dis_, and dNIC may be a new method for differentiating malignant SPNs from benign ones.

## 1. Introduction

Solitary pulmonary nodules (SPNs) which are defined as the isolated, round, or oval areas of increased opacity less than or equal to 3cm in diameter, surrounded by lung parenchyma, should not be associated with atelectasis, pulmonary hilar enlargement, or pleural effusion [1–3]. The diagnosis and management of solitary pulmonary nodule are a common and costly challenge in medicine [4, 5]. Conventional CT plays an important role in capturing morphological and enhancement features of SPNs. However, there is considerable overlap in the features between malignant and benign SPNs that lead to an erroneous diagnosis of pulmonary nodules [6, 7]. Therefore, research is being directed towards the development of noninvasive functional imaging techniques, such as dual-energy spectral CT [8, 9].

Gemstone spectral imaging (GSI) is a novel introduced technique based on the rapid switching between high- and low-energy data sets from view to view during a single rotation on the high-definition GE Discovery CT750 HD scanner, which provides more analyzing tools and quantitative parameters to help distinguish the malignant SPNs from the benign ones [8, 10]. It could enable the generation of material decomposition images that allows one to measure iodine component on iodine-enhanced images, and this is considered to be comparable to the real value of enhancement. Iodine concentration on iodine-enhanced images generated from spectral CT reflects the blood supply of SPNs, which is an essential evidence for differential diagnosis [11–14]. The spectral CT had multiple clinical uses in diagnosing pulmonary nodules, pulmonary embolism, assessing noninvasively angiogenesis of advanced gastric cancer, and differentiating small hepatocellular carcinoma from small hepatic hemangioma [13–17].

According to the previous research, Zhao et al. had firstly reported a new phenomenon of SPN heterogeneity in PET/CT, indicating that there was significant difference in spatial distribution of ^18^F-FDG metabolism between malignant and benign pulmonary nodules [18–21]. It has been reported that there was correlation between the SUV on PET/CT imaging and the iodine concentration on spectral CT imaging [22], in reflecting the blood supply and vascular density of SPNs. As a follow-up research, we hypothesized that a similar phenomenon of SPN would be observed on the spectral CT, as on the PET/CT. To our best knowledge, no clinical studies have directly analyzed the iodine concentration spatial distribution difference of SPN using spectral CT to differentiate malignancy and benignancy. Therefore, the purpose of this study was to explore the predictive value of spatial distribution difference in iodine concentration in SPNs with spectral CT.

## 2. Materials and Methods

The local institutional review board approved this prospective study, and written informed consent was obtained from all patients (no. 2017-152).

### 2.1. Patients

From July 2015 to June 2016, a total of 68 consecutive patients with suspected solitary pulmonary nodules were recruited, which were identified on CT scans obtained at local hospitals and/or clinics.

The patients who were selected for this study met the following criteria: (a) the presence of solid SPNs with the diameter more than 8 mm and less than 30 mm, (b) no contraindications to the administration of iodinated contrast material, and (c) having the ability to participate in the procedures cooperatively. Diameter was defined as the maximum diameter on the conventional thin-section CT scan with a lung window setting. The patients with ground glass nodules and/or nonsolid nodules were excluded on conventional thin-section CT scans. Ten patients were subsequently excluded: nine patients were excluded owing to the absence of pathologic diagnosis; one patient was excluded owing to noncooperation with the procedures. In the study, the follow-up CT of one patient was performed for 4 months, and the follow-up CT of other patient was performed for 6 months. So they were excluded from our research. As we known, if a nodule demonstrates a stable size for more than 2 years at comparison with prior radiographs, it has a high likelihood of being benign, and no further assessment is recommended [23]. In our study, a nodule demonstrates a stable size for more than 2 years at comparison with prior radiographs (n=1); two nodules of the patient were disappeared after anti-inflammatory therapy (n=2).

Thus, 60 consecutive patients in total (age range, 36-84 years; mean age, 61.4±10.3 years, 40 men and 20 women) were included to this study. Sixty SPNs (mean diameter 18.03±5.77mm; range, 8-30mm) were eventually included in the data analysis. All the patients underwent spectral CT or tracheal-bronchial biopsy (transbronchial or percutaneous) or bronchoalveolar lavage or microbiological examination or nodule resection by video-assisted thoracic surgery or conventional follow-up CT. For all nodules, final diagnoses were subsequently confirmed by a video-assisted thoracic surgery (n=36), CT-guided percutaneous or transbronchial biopsy (n=19), bronchoalveolar lavage (n=2), and clinically follow-up CT (n=3).

### 2.2. Groups

The SPNs were divided into two groups based on the final diagnosis. The malignant group (n=39, mean diameter, 19.26±5.99mm; range, 9-30mm) was composed of adenocarcinoma (n=33), squamous cell carcinoma (n=3), small cell carcinoma (n=1), and metastatic lung tumors (n=2). The benign group (n=21, mean diameter, 15.76±4.85mm; range, 8-28mm), included hamartoma (n=4), granuloma (n=2), tuberculoma (n=2), organizing pneumonia (n=11, comprising 10 organizing pneumonias confirmed with histological examination and 1 organizing pneumonia with their size unchanged for more than 2 year), and pulmonary inflammation (disappeared after anti-inflammatory therapy,n=2).

### 2.3. Spectral CT Scans

Three-phase contrast-enhanced chest scans were performed on a spectral CT scanner (GE Discovery CT750HD HDCT; GE Healthcare, Milwaukee, WI, USA) using GSI mode and low dose protocols in all the patients. Spectral imaging was obtained at 15 s (pulmonary artery phase, PP), 30 s (artery phase, AP), and 60 s (venous phase, VP) after contrast medium injection, respectively. The nonionic contrast media Ioversol Injection (320 mg I/ml, GE Healthcare) at the dose of 1.1 ml/kg was injected by using a pressure injector at a rate of 3.0 ml/s with antecubital venous access. Then, it was followed by 20 ml saline at the same rate. The scanning parameters included dual kVp (80 and 140 kVp); tube current 275 mA; scan range from apex to base of the lung; helical pitch 1.375:1; rotation speed of 0.7 s; detector coverage of 40 mm; field-of-view(FOV) 36 cm; section thickness of 0.625 mm; reconstruction interval of 0.625 mm. The CT dose index volume was a value of 7.64 mGy for each phase with spectral CT acquisition (comparable to 7.48±1.22 mGy dose administered for each phase of conventional contrast-enhanced scanning in a normal-size patient) at our institution.

### 2.4. Image Analysis

All the raw data were automatically reconstructed into images with slice thickness of 0.625 mm and spacing of 0.625 mm, and then the images were transferred to an AW4.6 workstation (GE Volume Share 6 AW4.6; GE Healthcare) for analysis with the use of GSI Volume Viewer software package. Iodine-based and water-based material decomposition images were obtained. Blinded to patient information and pathologic diagnosis, two radiologists (K.T. and L.Y.W, with 11 and 5 years of experience in chest CT, respectively) interpreted the CT images and measured the quantitative parameters in a workstation (ADW4.6; GE Healthcare) independent of each other, and their disagreement on measurement was resolved by consensus.

**Figure 1:**
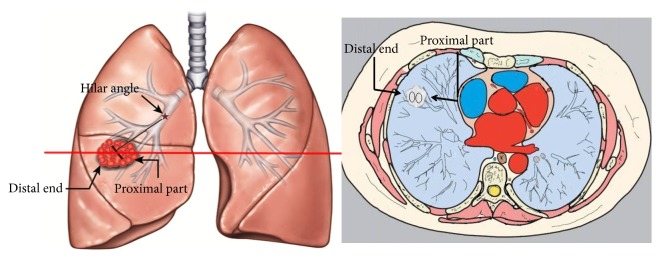
Illustration of proximal part and distal end of solitary pulmonary nodules in our study. The ipsilateral hilar angle and the direction of lung marking were the reference point.

We defined the area close to the ipsilateral hilar as the proximal region of SPNs and the area away from the ipsilateral hilar as the distal region (Figure 1). The region was divided along the direction of the lung marking. The iodine concentration (IC_les_) in both proximal and the distal regions of SPNs on the three phases of enhanced images were measured. The chest radiologist placed a circular ROI in as large an area as possible within the SPN. All ROI measurements were measured at three successive levels and averaged. The size, shape, and position of ROIs were the same between different phases by using the copy-and-paste function. For these measurements, a circular region of interest (ROI) that avoided cavity, calcification and blood vessels were placed at specific area in SPNs, and the selected ROI on images of one phase was identical with the other two phases. The concentration of iodine (IC_ao_) in aorta descendens corresponding to each ROI in lesion was also measured. Normalized iodine concentrations (NIC) were calculated as the ratio of iodine concentration in lesion and aorta descendens (NIC=IC_les_/IC_ao_), and the difference in normalized iodine concentration between the proximal and the distal regions was archived (dNIC=NIC_pro_-NIC_dis_). Moreover, in the same slice, the mean iodine concentration (IC) in the entire region of lesion and the standard deviation (SD) of iodine concentrations were also measured. Moreover, two experienced radiologists on conventional CT images performed visual assessment.

### 2.5. Statistical Analysis

Commercial statistical analysis packages (version 20.0 SPSS, IBM) were used to analyze the measurements. All the data tested for normality. Mann–Whitney U-test was used to statistically compare these parameters at PP between malignant and benign nodules because data in this group did not meet the normality. The two-sample t-test was used to statistically compare these parameters at AP and VP. Receiver operating characteristic (ROC) analyses were used to compare the capability of NIC_pro_, dNIC in all three phases and visual assessment to distinguish malignant and benign SPNs. The threshold values of multiple parameters were determined by using of the ROC curves to optimize both the sensitivity and the specificity (Youden's index). P value <0.05 was considered to indicate a significant difference.

## 3. Results

Intragroup observer agreement (ICC) was used to assess the interobserver (K.T. and L.Y.W) reliability and agreement. Intragroup observer agreement (ICC) was significantly good and overall intragroup correlation coefficient in NIC_pro_ (ICC_pro_) and NIC_dis_ (ICC_dis_) was 0.810 and 0.849 at PP, 0.858 and 0.786 at AP, and 0.827 and 0.796 at VP (all P<0.001).

The data were expressed as mean ± SD or median and interquartiles (P50, P25, and P75). The measurement data of NIC_pro_, NIC_dis_, and dNIC at PP, AP, and VP of all the 60 cases were summarized in Table 1. There were significant differences in NIC_pro_ and NIC_dis_ between malignant and benign nodules in AP (mean NIC_pro_, 0.283 ±0.165 versus 0.183±0.081; P = 0.012; mean NIC_dis_ 0.190 ±0.117 versus 0.258 ±0.094; P=0.024) and VP (mean NIC_pro_, 0.577±0.199 versus 0.407±0.247; P=0.005; mean NIC_dis_ 0.406±0.142 versus 0.591±0.247; P =0.004). Significant differences were also found in NIC_pro_ at PP (median and interquartiles (0.67, 0.41, 1.19 versus 0.38, 0.19, 0.82); P = 0.037). However, no significant differences were found in NIC_dis_ at PP (median and interquartiles (0.43, 0.22, 0.64 versus 0.53, 0.36, 1.29); P=0.093). Moreover, significant higher dNIC values in the proximal regions were found in malignant nodules than benign nodules at PP (median and interquartiles (0.31, 0.11, 0.57 versus -0.26, -0.5, -0.1); p≤0.001), AP (mean dNIC, 0.093 ±0.094 versus -0.075±0.060; p≤0.001), and VP (mean dNIC, 0.171±0.137 versus -0.183± 0.127; p≤0.001).

Quantitative assessment of IC and SD of IC at PP, AP, and VP was showed in Table 2. No differences were found in IC between malignant and benign nodules in all three phases. However, SD of IC in malignant nodules was higher than that in benign nodules at AP (malignant: 9.701±2.922, benign: 8.245±1.796, P = 0.020).

**Figure 2:**
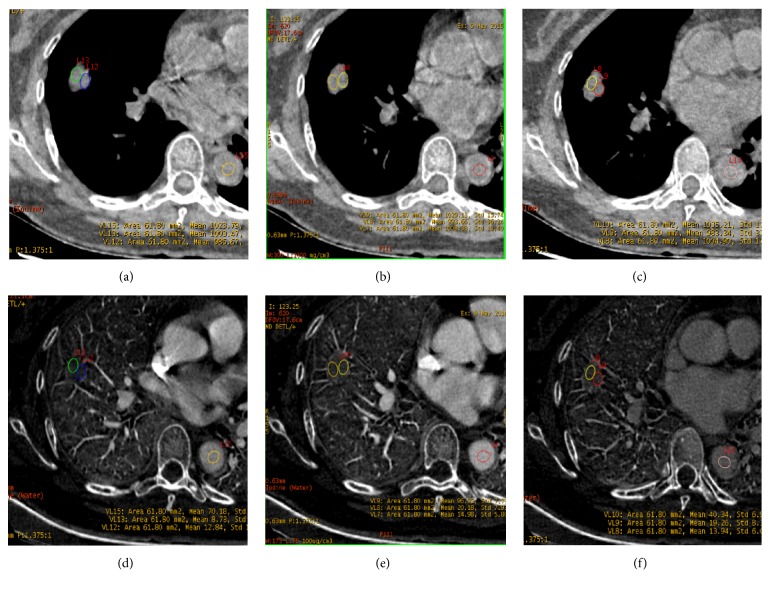
A 58-year-old woman with adenocarcinoma in the middle lobe of right lung. The spectral CT imaging (section thickness, 0.625 mm) water-based material decomposition images (a-c) and iodine-based material decomposition images (d-f) at PP, AP, and VP. The region of interest (ROI) was drawn in the proximal and distal area of the lesion. dNIC (PP) =0.06, dNIC (AP) = 0.05, and dNIC (VP) = 0.13.

**Figure 3:**
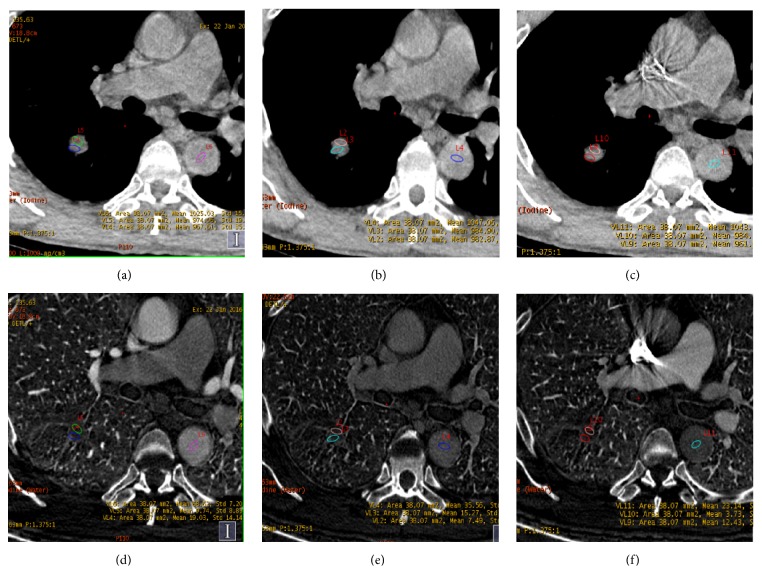
A 60-year-old man with tuberculosis in the inferior lobe of right lung. The spectral CT (section thickness, 0.625 mm) water-based material decomposition images (a-c) and iodine-based material decomposition image (d-f) at PP, AP, and VP. The region of interest (ROI) was drawn in the proximal and distal area of the lesion. dNIC (PP) = -0.38, dNIC (AP) = -0.14, and dNIC (VP) = -0.22.

**Figure 4:**
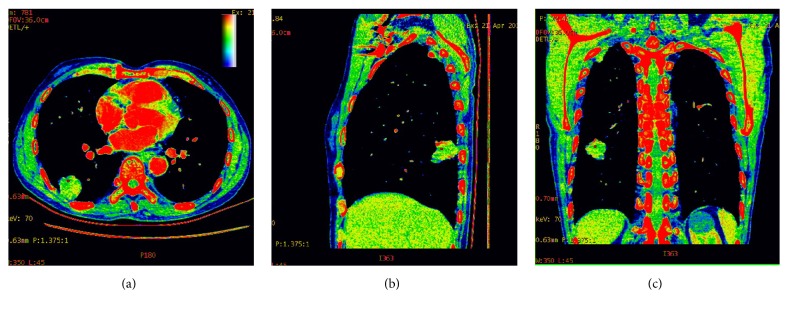
An 80-year-old man with adenocarcinoma in the inferior lobe of right lung. Pseudocolor images of spectral CT on the artery phase can visually display distribution difference of iodine concentration based on blood supply in tumor by different colors. The color in proximal region was brighter than that of distal one.

**Figure 5:**
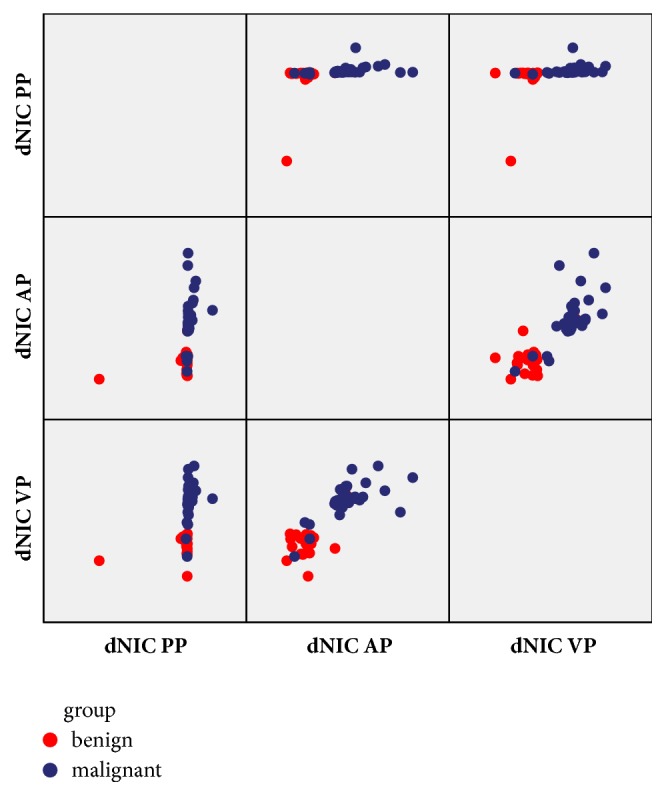
The three-dimensional scatter plots of dNIC between malignant and benign nodules in three phases. From the scatter plot, the dNIC distribution of malignant and benign nodules reflects distribution difference of iodine concentration.

Figures 2 and 3 showed images in two patients (one with pulmonary adenocarcinoma and the other one with tuberculosis). Pseudocolor images generated from the artery phase, which visually display heterogeneity on blood supply in tumor by different colors, were showed in Figure 4. The scatter plot of dNIC between malignant and benign nodules in three phases was displayed in Figure 5

**Figure 6:**
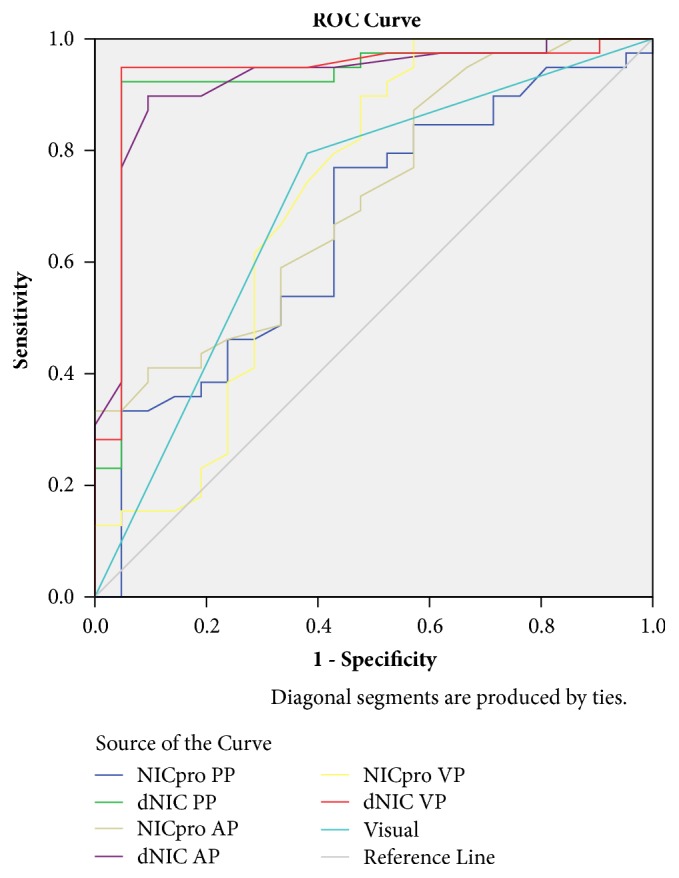
Receiver operating characteristic (ROC) curves by using NIC in the proximal region and dNIC to differentiate lung cancers from benign nodules during pulmonary arterial phase (PP), arterial phase (AP), and venous phase (VP). dNIC, the NIC value difference between the proximal and distal regions of nodules; NIC, normalized iodine concentration; ROC, receiver operating characteristic.

The ROC curves for predicting malignant and benign nodules based on NIC_pro_ and dNIC in the three phases and visual assessment were shown in Figure 5. The AUC, based on NIC at VP (AUC = 0.933), was greater than those based on other quantitative parameters (Figure 6; Table 3). The diagnostic sensitivity and specificity of NIC_pro_ and dNIC and visual assessment were as follows: NICpro: 77% and 57% with threshold of 0.40 in PP, 67.0% and 57% with threshold of 0.47 in AP, and 89% and 52% with threshold of 0.36 in VP; dNIC: 92% and 95% with threshold of -0.35 in PP, 89% and 90% with threshold of 0.00 in AP, and 93% and 95% with threshold of -0.07 in VP; visual assessment: 79% and 62% (Table 3).

## 4. Discussion

Nowadays, the detection rate of SPN can be up to 51% with the widespread use of multidetector CT (MDCT). Most of the detected SPNs have benign causes; however, there are still 20–30 % of them which are malignant, which are mainly in the early stage of lung cancer [24]. Lung cancer, the leading cause of cancer death throughout the world, is responsible for an estimated 158,040 deaths in 2015 [25]. Overall 5-year survival rates in patients with lung cancer remains rather low at 15%; however, stage IA of lung cancer that is treated with resection may portend a long-term survival of 80% [24]. Therefore, it is essential of the differentiation and treatment for the pulmonary nodules of strongly suspicious malignancy. However, it remains challenge of differential diagnosis because of the overlap in imaging features between malignant and benign nodules. Hence, the methodology of imaging has been developing from traditional morphological examinations to dynamic functional examinations.

Different techniques to obtain dual-energy data have been proposed and pursued by various vendors: rapid kVp switching (GE Healthcare, Milwaukee, WI); energy-sensitive sandwich detectors (Philips Medical Systems, Cleveland, OH); and dual-source CT (Siemens Healthcare, Forchheim, Germany). The system in our study was rapid kVp switching (GE Healthcare, Milwaukee, WI). Spectral CT can realize rapid alternation (in the order of 1000 times per second) of the tube potentials between, for instance, 80 and 140 kv during the gantry rotation [10, 26]. At present, dual-energy CT equipment used in clinical practice is spectral CT and dual-source CT. As a functional imaging method, spectral CT provides multiple parameters by a rapid kVp switching technique, such as material decomposition imaging, and it has the potential value of application in neoplastic diseases [11–13, 27].

According to our research, iodine concentrations in malignant nodules were lower than that in benign ones. The reasons may be that in the benign nodules, inflammatory nodules (n=15, including 11 with focal-organizing pneumonia, 2 with granulomatous inflammation, and 2 with pulmonary inflammation) account for about 71% (15/21) in our research. In previous study, patients with inflammatory masses also had significantly higher normalized iodine concentration values than patients with lung cancers. Because granulomatous inflammation and organizing pneumonia are formed by proliferation of inflammatory granulation tissue or residual of acute inflammation, the rich and dilatate capillaries of masses are stimulated by inflammation gradually [28]. The earlier studies had also shown that iodine concentration of spectral CT was positively correlated with the expression of VEGF in NSCLC [29]. In the spectral imaging, iodine and water were selected as the basis pair for material decomposition image, because that iodine, being the main ingredient of contrast medium, directly reflects the number of supplying vessels and blood flow in the SPNs [14, 28, 29].

Our results against spectral CT were consistent with the study of Zhao et al. against PET/CT [18]. There were significant differences in the NIC between malignant and benign SPNs, with respect to both of the proximal and distal region at AP and VP. In addition, the NIC of malignant SPNs was significantly higher in the proximal regions than that in the distal regions. Based on pathologic analysis, these observations might be associated with blood supply of SPN. As we all know, cancer tends to grow towards the blood vessels to find nutrition [30]; thus the proximal region of malignant nodules was usually rich in blood supply. After enhanced CT scan, high-density iodine is the main media material in the blood vessel, so the iodine concentration in the ROI directly reflects its blood supply. However, no significant differences were found in iodine concentration between malignancy and benignancy (malignant versus benign: P = 0.089, 0.633, 0.619, respectively, in PP, AP, and VP). In addition, our results clearly showed that the SD of iodine concentration of malignant nodules was higher than that of benign ones. As far as we know, malignant tumors showed much more inhomogeneous enhancement than benign ones. The establishment of angiogenesis and vascular networks usually occur in malignant tumor, which could not support the rapid growth of neoplasm, and the subsequent reduction in the delivery of oxygen renders lung cancer hypoxic. Moreover, the difference of SD was statistically significant in VP (P=0.020). In other words, the spectral CT in VP can better show the distribution difference of iodine concentration of SPN by SD of iodine concentrations.

The present study has some limitations. First, the study population was relatively small in our study, necessitating larger prospective studies for confirmation. Second, volumetric measurements of the iodine concentration should be measured in study. Instead of volumetric measurement, all ROI measurements were measured at three successive levels and averaged in our research. Further clinical trials need to be performed in the future. Last, data could be limited to the single-source platform used and may not be applicable to the dual-source CT system. As we know, both rapid kilovoltage switching single-source spectral CT and detector-based dual-source CT can perform two-material decomposition analysis such as iodine- and water-based decomposition from high- and low-energy projection [31]. On the dual-source CT system, it is quite expected that the data will also be applicable for dual-source CT, but it need further study to confirm.

In conclusion, the difference of iodine concentration spatial distribution related to blood supply in SPNs helps for differentiation of malignancy and benignancy on contrast-enhanced spectral CT by analyzing multiple quantification parameters. In addition, SD of iodine concentrations in venous phase was found to reflect distribution difference of iodine concentration better. Quantitative analysis of iodine concentration spatial distribution difference based on blood supply by spectral CT may be a promising new method for differentiating malignant and benign pulmonary nodules and has the potential application values in future.

## Figures and Tables

**Table 1:** Quantitative assessment of NIC and dNIC in the proximal and distal regions of malignant and benign nodules in PP, AP, and VP.

Group	Malignant nodules	Benign nodules	P value
IC_PP_	11.128±6.509	14.381 ±7.010	0.089
IC_AP_	18.410±8.497	19.520 ±8.675	0.633
IC_VP_	17.440±5.637	18.620 ±9.952	0.619
SD_PP_	8.1723±2.176	7.891±1.297	0.591
SD_AP_	9.701±2.922	8.245±1.796	0.020
SD_VP_	8.457±2.292	7.535±1.426	0.060
Longest diameter (mm)	19.26±5.999	15.76±4.847	0.018

PP, pulmonary phase; AP, arterial phase; VP, venous phase; IC: iodine concentration; SD: standard deviation of iodine concentration in all nodules.

**Table 2:** Quantitative assessment of IC and standard deviation of iodine concentration in PP, AP, and VP.

Group	NIC_pro_	NIC_dis_	dNIC
PP			
Malignant nodules	(0.67,0.41, 1.19)	(0.43, 0.22, 0.64)	(0.31, 0.11, 0.57)
Benign nodules	(0.38, 0.19, 0.82)	(0.53,0.36,1.29)	(-0.26, -0.5, -0.1)
P value	0.037	0.093	p≤0.001
AP			
Malignant nodules	0.283 ±0.165	0.190 ±0.117	0.093 ±0.094
Benign nodules	0.183 ±0.081	0.258 ±0.094	-0.075 ±0.060
P value	0.012	0.024	p≤0.001
VP			
Malignant nodules	0.577±0.199	0.406±0.142	0.171±0.137
Benign nodules	0.407±0.247	0.591±0.247	-0.183±0.127
P value	0.005	0.004	p≤0.001

PP, pulmonary phase; AP, arterial phase; VP, venous phase; NIC_pro_, normalized iodine concentration in the proximal region; NIC_dis_, normalized iodine concentration in the distal region; dNIC, NIC_pro_-NIC_dis_.

**Table 3:** Diagnostic indices of NICpro, dNIC in PP, AP, and VP, and other methods for differentiating malignant and benign SPNs.

Criteria	AUC	Sensitivity	Specificity	TP	FP	FN	TN	PPV	NPV (%)
		(%)	(%)					(%)	
NICpro(PP)>0.40	0.644	77	57	26	13	13	8	67	38
NICpro(AP)>0.47	0.716	67	57	3	0	36	21	100	37
NICpro(VP)>0.36	0.714	89	52	35	10	4	11	78	73
dNIC(PP) >-0.35	0.923	92	95	37	13	1	9	74	90
dNIC(AP) >0.00	0.924	89	90	35	2	4	19	94	82
dNIC(VP) >-0.07	0.933	93	95	37	1	2	20	97	90
Visual assessment (conventional CT)	0.707	79	62	31	8	8	13	79	62

## Data Availability

The Excel data used to support the findings of this study are available from the corresponding author upon request.
